# Polymicrobial vertebral osteomyelitis after oesophageal biopsy: a case report

**DOI:** 10.1186/s12879-016-1471-9

**Published:** 2016-03-31

**Authors:** Aude Giger, Erlangga Yusuf, Oriol Manuel, Olivier Clerc, Andrej Trampuz

**Affiliations:** Infectious Disease Service, Department of Medicine, Lausanne University Hospital (CHUV), Rue du Bugnon 46, CH-1011 Lausanne, Switzerland; Laboratory Medicine, Gasthuiszusters Antwerpen Hospital, Antwerpen, Belgium; Department of Medicine, Neuchâtel cantonal Hospital, Neuchâtel, Switzerland; Center for Musculoskeletal Surgery, Charité University Medicine Berlin, Berlin, Germany

**Keywords:** Spondylodiscitis, Postinterventional osteomyelitis, Mixed infections

## Abstract

**Background:**

While most cases of polymicrobial vertebral osteomyelitis are secondary to hematogenous seeding, direct inoculation during spinal surgery and contiguous spread from adjacent soft tissue are also potential routes whereby pathogens may infect the spine.

**Case presentation:**

A 74 year-old man presented with an exacerbation of back pain after a fall. His past medical history included hepatocellular and oesophageal carcinoma. Three months earlier he had undergone an endoscopic biopsy of the oesophagus for routine follow-up of his oesophagus carcinoma. He also underwent a vertebroplasty due to suspected pathologic fracture. On admission to hospital, magnetic resonance imaging revealed an infiltrative process at the level of the 5th and 6th thoracic vertebrae. Blood cultures were positive for both *Streptococcus mitis* and *Gemella morbillorum*. During his course of antibiotic therapy he developed an abscess at the level of 8th thoracic vertebrae and culture of this abscess grew *Candida albicans*. He was treated with antibiotics and antifungal drugs and recovered fully.

**Conclusion:**

Vertebral osteomyelitis may be caused by direct spread following an oesophageal procedure. Microbiological diagnosis is essential to target the specific pathogen, especially in cases of polymicrobial infection.

## Background

Vertebral osteomyelitis, also known as spinal osteomyelitis, spondylodiscitis, septic discitis, or disk-space infection [1] is a rare disease; its incidence is estimated at 2.4 cases per 100,000 persons [2]. Most cases results from hematogenous seeding from a distant source such as the urinary tract, skin, infected vascular catheters, endocarditis, or bursitis/septic arthritis [3, 4]. Other routes of infection include direct external inoculation and spread from contiguous soft tissues. Predisposing factors include diabetes mellitus, advanced age, intravenous drug use, immunosuppression, malignancy, renal failure, and rheumatological disease [5]. Vertebral osteomyelitis is primarily a monomicrobial bacterial infection; predominant pathogens are *Staphylococcus aureus*, coagulase-negative staphylococci (in cases where a foreign body or hardware is present), streptococcal species and enterobacteriaceae [2]. Polymicrobial infections occur in less than 10 % of cases and are most likely to result from contiguous spread [6].

We describe here a unique case of polymicrobial vertebral osteomyelitis following an oesophageal biopsy which was originally misdiagnosed as a bone metastasis.

## Case presentation

A 74 years old male was admitted to the internal medicine department due to an acute exacerbation of his chronic back pain following a fall in his bathroom the previous night. He also reported an involuntary weight loss of 15 kg. His past medical history included hepatocellular carcinoma four years ago and an epidermoid carcinoma of the upper oesophagus five years ago. Both types of carcinoma were considered in remission at the time of his admission to hospital. He had been on oral dexamethasone for 2 weeks for his back pain and sorafenib as maintenance chemotherapy for his oesophagus carcinoma.

**Fig. 1:**
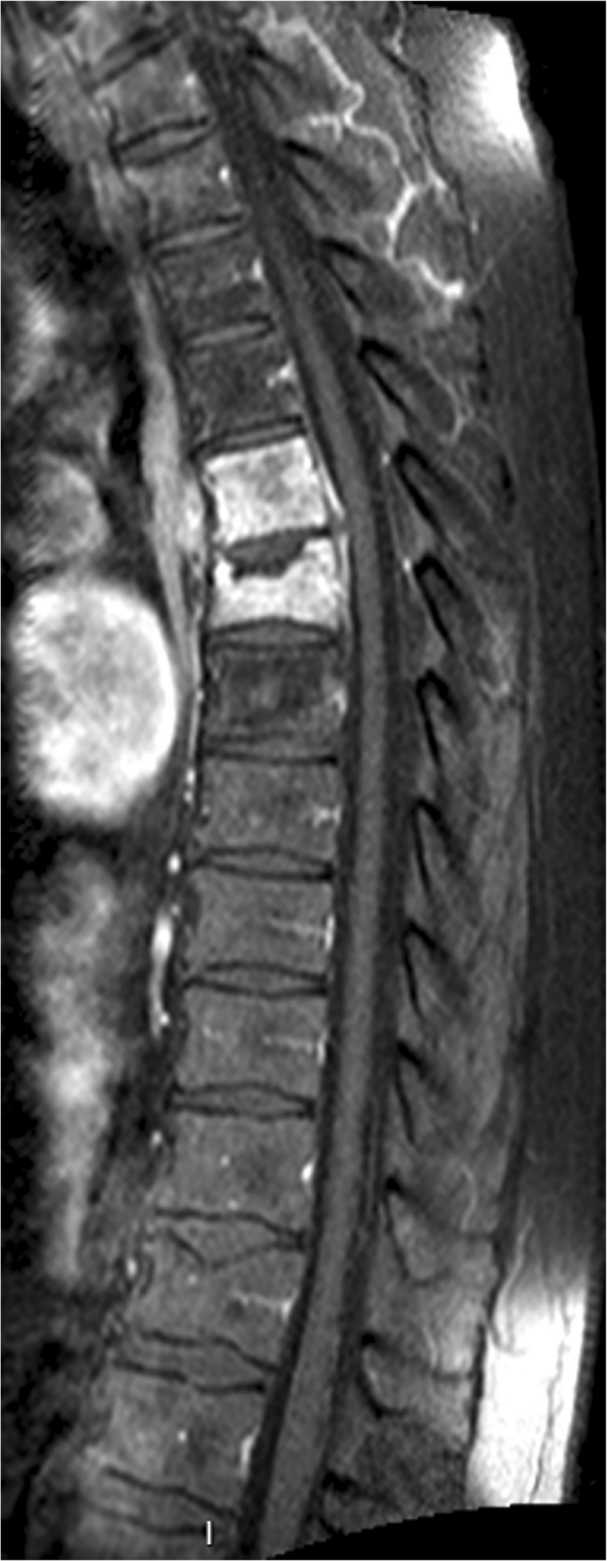
Spine MRI shows fracture settlement of the sixth thoracic vertebra with infiltration from the fifth to the sixth thoracic vertebra

The patient reported that his back pain had started three months before the current hospitalisation without history of trauma. A magnetic resonance imaging (MRI) scan was performed (Fig. 1) which demonstrated pathologic infiltrations of the 5th and 6th thoracic vertebra without involvement of the intervertebral disc, and associated with a fracture of the upper plate of the 6th thoracic vertebra. Bony metastasis with pathologic fracture was suspected and a transcutaneous vertebroplasty was performed by injecting high-viscosity polymethylmethacrylate cement. The bone biopsy taken prior to this procedure showed signs of acute and chronic osteomyelitis without sign of malignancy. The Gram stain did not reveal the presence of bacteria, and bacteria culture of the biopsy was negative.

On physical examination at admission the patient appeared ill with a temperature of 38.3 °C. Auscultation of the heart and lungs was unremarkable. The 7th thoracic vertebra was painful on palpation. Laboratory tests revealed an increased C-reactive protein level (41 mg/l, normal: <10 mg/l) and an increased leukocyte count (20.3 G/l, normal: 4 to 10 G/l). Blood cultures grew *Streptococcus mitis* and *Gemella morbillorum* in 4 out of 6 bottles, both pathogens susceptible to clindamycin (MIC of 0.064 ug/ml for *S.mitis* and 0.094 ug/ml for *G.morbillorum*) and moxifloxacin (MIC of 0.25 ug/ml for *S.mitis* and 0.25 ug/ml for *G.morbillorum*).

Further information regarding the timeline of symptoms was obtained; his back pain started suddenly a few days after an endoscopic biopsy of the oesophagus had been performed, three months earlier, as follow-up for his oesophagus carcinoma. The oesophageal biopsy was negative. Thoracic and abdominal computed tomography (CT) scan images obtained one month before his admission showed no radiological signs of malignancy. This new information lead us to suspect of polymicrobial vertebral osteomyelitis by direct inoculation with a secondary bacteriemia due to recent bone sampling. Treatment with intravenous amoxicillin/clavulanate (2.2 g every 8 h) was initiated, and switched 48 h later to intravenous ceftriaxone (2 g once daily).

**Fig. 2:**
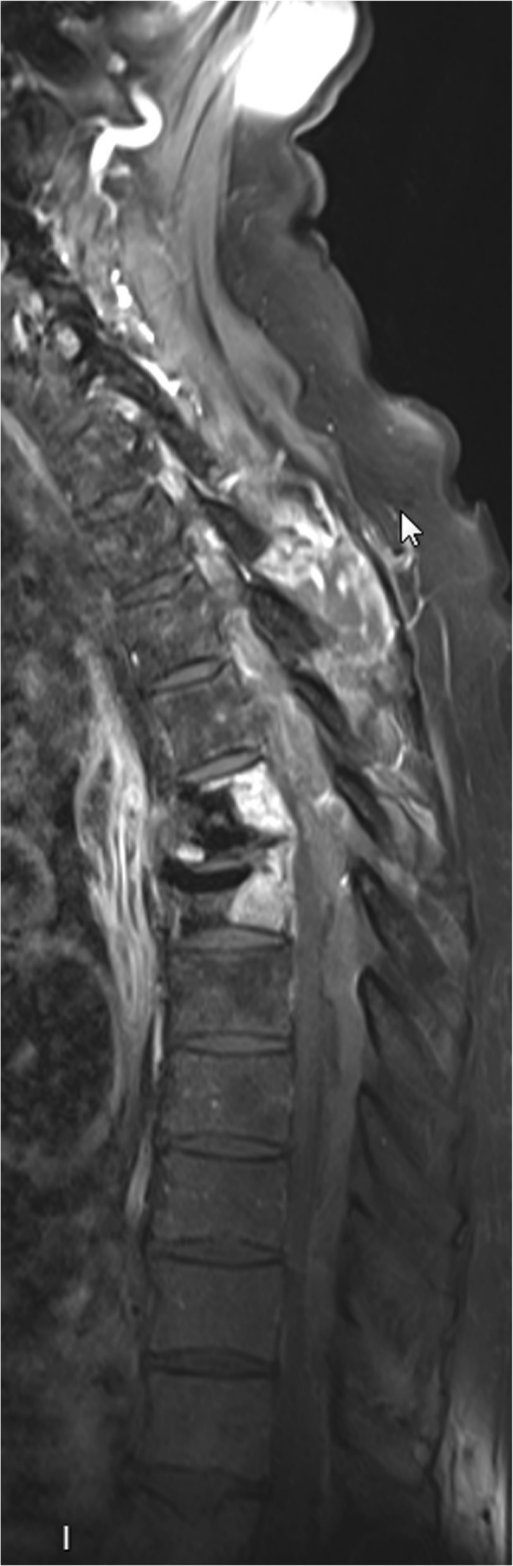
Spine MRI performed after Gadolinium intravenous contrast shows epidural posterior abscess from the third cervical (C3) to the first lumbar vertebra (L1). Medullary compression is seen without medullary suffering. Not well shown in this figure is abscess around the cement (*fifth* and *sixth*) thoracic vertebra

A transthoracic echocardiogram was performed, and did not show any valvular abnormality. A CT scan of the chest revealed thickening of oesophageal wall with oedema, compatible with an oesophageal perforation. Accordingly, an oesophageal endoscopy was performed which demonstrated thickening of posterior oesophageal wall at the same level as the vertebroplasty. An MRI of the spine demonstrated an epidural abcess from the 3rd cervical to the 1st lumbar vertebra with minimal medullary compression (Fig. 2).

**Fig. 3:**
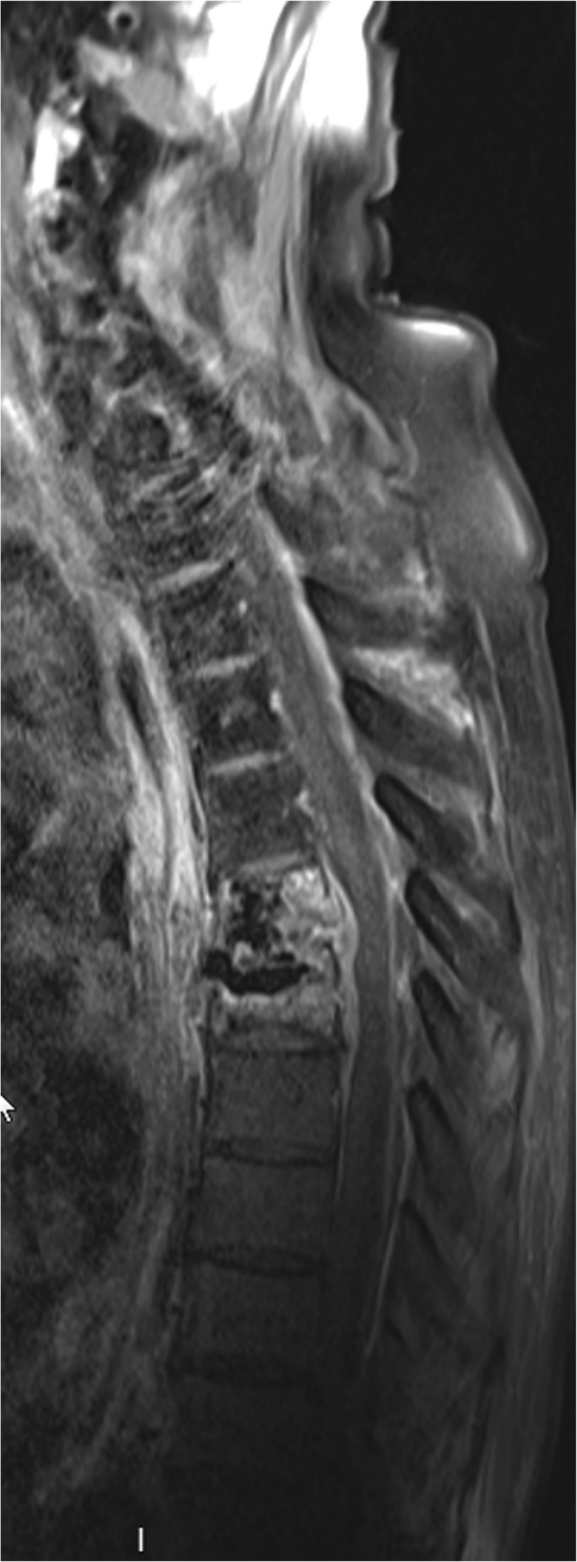
Spine MRI shows improvement from earlier imaging, with decrease of epidural abcess and of medullary compression. Resolution of the abscess surrounding the site of cementoplasty as well as anterior abscess at the level of third thoracic to six thoracic and the soft tissue abcess

**Fig. 4:**
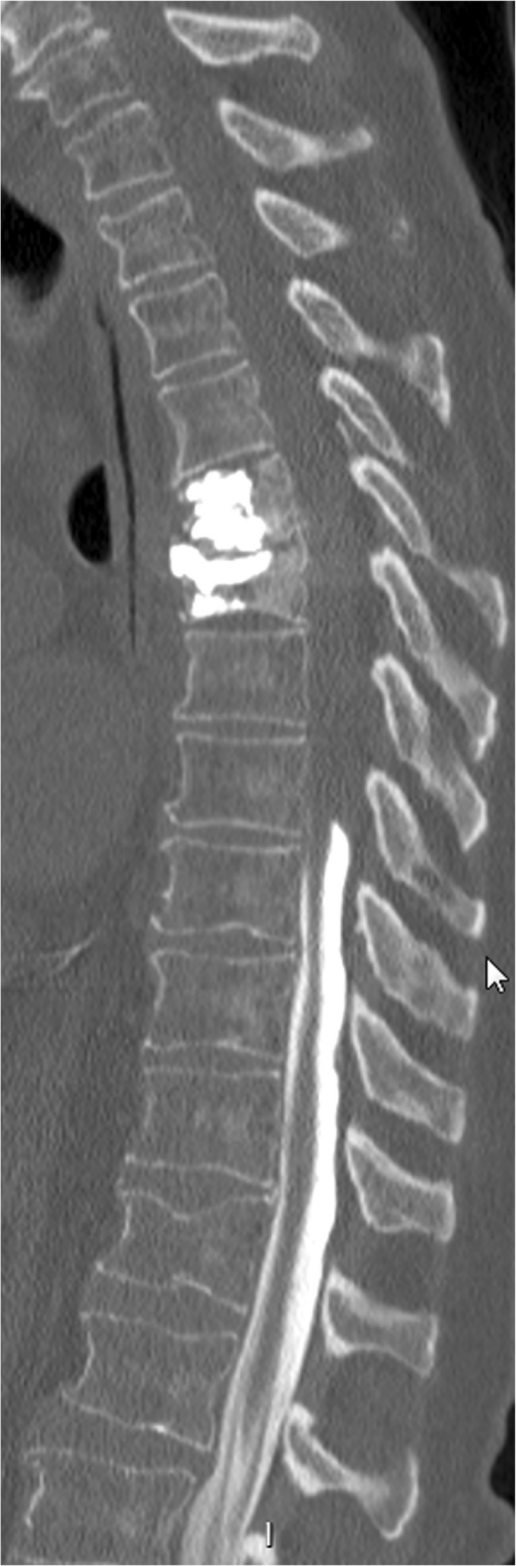
Spine CT shows a block from the level of eight thoracic vertebrae due to an epidural posterior mass

No surgical procedure was performed given the lack of neurological signs, and antibiotic treatment was continued. Clinical evolution was favourable and an MRI performed three weeks after initiation of antibiotic therapy showed regression of the epidural abscess (Fig. 3). Intravenous antibiotics were switched after six weeks to oral moxifloxacin (400 mg once daily), with a planned course of three months. Two days after initiation of oral antibiotic therapy the patient developed weakness of the right lower limb with progressive loss of sensation, extending to the left lower limb within 24 h. A CT of the spine revealed a new epidural abscess at the level of the 8th thoracic vertebra (Fig. 4). Intravenous ceftriaxone and metronidazole were reintroduced and a laminectomy at the 7th and 8th thoracic vertebra was immediately performed. The abscess was cultured and grew *Candida albicans*. Intravenous caspofungin was added and subsequently replaced by oral fluconazole (400 mg once daily) after two weeks. Metronidazole and ceftriaxone were switched to moxifloxacin after four weeks, and then to clindamycin due to a prolonged QT interval on the electrocardiogram. Therapy with clindamycin was maintained for three months and fluconazole for six months. The patient’s symptoms improved gradually, and an MRI performed twelve weeks after admission to our hospital revealed no residual abscess. At 6 month follow-up he had recovered almost fully, with only slight motor weakness of lower limbs.

## Discussion

We describe a case of polymicrobial vertebral osteomyelitis initially misdiagnosed as a bony metastasis. This particular case is one of few cases of vertebral osteomyelitis that have been described after an oesophageal biopsy [7–10]. Cases of post-vertebroplasty osteomyelitis are also rare in the literature [11].

When vertebral osteomyelitis is suspected, imaging of the spine followed by percutaneous bone biopsy should be performed. If polymicrobial osteomyelitis is suspected, a biopsy should be performed regardless of blood culture results [1] and immediate empirical antibiotic therapy should be initiated to cover the most common agents such as Staphylococci, Streptococci and enteric Gram negative bacilli (for example amoxicillin/clavulanate or a first or second generation cephalosporin, with or without vancomycin, depending on the local prevalence of MRSA). This may halt the progression of bony destruction and prevent the need for surgical treatment [12]. When vertebral collapse and spinal cord compression occur, surgical debridement and stabilisation should be combined with medical therapy to eradicate infection and resolve neurological deficits.

We hypothesize that the vertebral osteomyelitis in this case was likely caused by direct spread after oesophageal biopsy for two reasons: the posterior wall thickening seen at the time of the 2nd endoscopy at the same level as the vertebral infiltrate is consistent with a remote healed perforation. In addition, the polymicrobial flora, including *Candida albicans*, is suggestive of an upper gastrointestinal tract origin.

Candidal infections are most commonly described in immunocompromised hosts (corticosteroids and sorafenib in this case) and in patients receiving prolonged courses of antibacterial therapy. In addition to direct spread from the oesophagus, *Candida albicans* may have been introduced during the vertebroplasty procedure or the Candidal infection may also have been a superinfection favoured by prolonged antibiotic therapy [13]. Vertebroplasty is, in general, a safe procedure but cases of osteomyelitis have been described [3, 4]. The cement used may have acted as a biofilm and could explain the slow progression of the infection.

There are several reasons for the delayed diagnosis of vertebral osteomyelitis in our patient. Firstly, due to patient’s past medical history, clinical presentation and MRI images, it was difficult to differentiate infection from neoplasia. Secondly, the recent vertebroplasty made performing a new bone biopsy difficult. Finally, the abscess did initially regress on MRI after three weeks of antibiotics, which was interpreted as an appropriate response to antibiotic therapy.

## Conclusion

In conclusion, we present here an uncommon case of vertebral osteomyelitis after oesophageal biopsy. Because antibiotic therapy was directed to the pathogens found in the blood, *Candida albicans* was not suspected as the cause of a clinical relapse. In cases of polymicrobial vertebral osteomyelitis, we suggest performing a bone biopsy to appropriately target antibiotic therapy, and to systematically look for potential contiguous sources of infection.

## Consent

Written informed consent was obtained from the patient for publication of this case report and any accompanying images. A copy of the written consent is available for review by the Editor of this journal.
